# Draft Genome Sequence of Brazilian *Leptospira noguchii* Serogroup Panama Strain U73, Isolated from Cattle

**DOI:** 10.1128/genomeA.01179-15

**Published:** 2015-10-15

**Authors:** Luisa Z. Moreno, Ana P. Loureiro, Fabiana Miraglia, Carlos E. C. Matajira, Frederico S. Kremer, Marcos R. Eslabao, Odir A. Dellagostin, Walter Lilenbaum, Andrea M. Moreno

**Affiliations:** a Laboratório de Epidemiologia Molecular e Resistência a Antimicrobianos, Faculdade de Medicina Veterinária e Zootecnia, Universidade de São Paulo, São Paulo, Brazil; b Departamento de Microbiologia e Parasitologia, Laboratório de Bacteriologia Veterinária, Universidade Federal Fluminense, Rio de Janeiro, Brazil; c Centro de Desenvolvimento Tecnológico, Núcleo de Biotecnologia, Universidade Federal de Pelotas, Pelotas, Brazil

## Abstract

*Leptospira noguchii* is a current zoonotic pathogen in Brazil. Here, we report the draft genome sequence of the Brazilian *L. noguchii* serogroup Panama strain U73, isolated from asymptomatic cattle urine.

## GENOME ANNOUNCEMENT

*Leptospira noguchii* is one of the pathogenic species of the *Leptospira* genus. It is widely described as a zoonotic pathogen in the Americas (1–5). In Brazil, *L. noguchii* has already been isolated from diseased humans and a dog (3) and also from asymptomatic sheep and cattle (4, 5). Although the epidemiology of *L. noguchii* infection has not yet been elucidated, its isolation from humans and livestock reinforces the importance of this species in the Brazilian leptospirosis scenario.

Here, we present the draft genome of *L. noguchii* serogroup Panama strain U73, isolated from asymptomatic cattle urine in Rio de Janeiro, Brazil. Isolation, serogrouping, and analysis of partial *rrs* and *secY* sequences of this strain were previously described by Martins et al. (5). Genomic DNA was extracted and purified with the illustra bacteria genomicPrep mini spin kit (GE Healthcare) and used for paired-end library preparation with the Nextera DNA sample prep kit (Illumina) and sequencing through the Illumina MiSeq platform.

Sequencing resulted in 789,252 paired-end quality filtered reads with approximately 50-fold coverage. The *de novo* assembly was performed with CLC Main Workbench version 7.5.1 (CLC Bio) and Geneious version 8.0.5 (Biomatters Ltd.) and resulted in 279 scaffolds with an *N*_50_ of 24,125. The U73 draft genome comprises ~4.76 Mbp, with an overall G+C content of 35.63%. Automatic genome annotation was performed with BASys (Bacterial Annotation System) (6).

In total, 4,535 coding sequences, 37 tRNAs, and 4 rRNAs were identified. In addition, ISFinder (7) was used for the prediction of insertion sequence (IS) elements; IS1500, IS1501, IS1502, and ISLin2 were identified in the U73 draft genome. Regarding the virulence factors, strain U73 presents genes encoding lipoproteins and immunoglobulin-like proteins, such as *lipL41*, *lipL36*, *lolC/D*, and *ligA*, which correspond to the main virulence factors of *L. interrogans*. Interestingly, several antimicrobial resistance genes were identified, including efflux pumps (*mdtA*, *mdtB*, *norM*), as well as metal resistance genes (*sugE*, *czcA*, *czcD*).

A multilocus sequence typing (MLST) analysis was also performed using the MLST server (8). Novel sequence types (STs) were identified for the U73 strain among the *Leptospira* MLST 2 and 3 schemes (ST97 and ST137, respectively). Despite the wide range of alleles and the strain’s incongruity between the schemes, the U73 strain presented greater proximity with *L. noguchii* strains from serogroups Autumnalis and Louisiana at MLST schemes 2 and 3, respectively. There are only two more *L. noguchii* serogroup Panama in the *Leptospira* MLST database; however, they are more distant from the U73 strain than the aforementioned serogroups.

Although being pathogenic, *L. noguchii* has been scarcely studied. Sequencing of new strains and the increase of genetic and epidemiological data on this *Leptospira* species will enhance the knowledge about *L. noguchii* infection, transmission, and control.

### Nucleotide sequence accession number.

This whole-genome shotgun project has been deposited in DDBJ/ENA/GenBank under the accession number LHQT00000000.
